# Enhanced Dendritic Cell-Mediated Antigen-Specific CD4+ T Cell Responses: IFN-Gamma Aids TLR Stimulation

**DOI:** 10.1155/2013/516749

**Published:** 2013-05-28

**Authors:** Kuo-Ching Sheng, Stephanie Day, Mark D. Wright, Lily Stojanovska, Vasso Apostolopoulos

**Affiliations:** ^1^Immunology and Vaccine Laboratory, Burnet Institute, Melbourne, VIC 3004, Australia; ^2^Institute for Glycomics, Griffith University, Gold Coast, QLD 4215, Australia; ^3^Department of Immunology, Monash University, Melbourne, VIC 3004, Australia; ^4^College of Health and Biomedicine, Victoria University, VIC 3021, Australia; ^5^VA Consulting Services, Melbourne, VIC 3030, Australia

## Abstract

Phenotypic maturation and T cell stimulation are two functional attributes of DCs critical for immune induction. The combination of antigens, including those from cancer, with Toll-like receptor (TLR) ligands induces far superior cellular immune responses compared to antigen alone. In this study, IFN-gamma treatment of bone marrow-derived DC, followed by incubation with the TLR2, TLR4, or TLR9 agonists, enhanced DC activation compared to TLR ligation alone. Most notably, the upregulation of CD40 with LPS stimulation and CD86 with CpG stimulation was observed in *in vitro* cultures. Similarly, IFN-gamma coinjected with TLR ligands was able to promote DC activation *in vivo*, with DCs migrating from the site of immunization to the popliteal lymph nodes demonstrating increased expression of CD80 and CD86. The heightened DC activation translated to a drastic increase in T cell stimulatory capacity in both antigen independent and antigen dependent fashions. This is the first time that IFN-gamma has been shown to have a combined effect with TLR ligation to enhance DC activation and function. The results demonstrate the novel use of IFN-gamma together with TLR agonists to enhance antigen-specific T cell responses, for applications in the development of enhanced vaccines and drug targets against diseases including cancer.

## 1. Introduction

Vaccination requires highly purified proteins or synthetic peptides usually in combination with immune stimulating adjuvants or danger signals, to successfully prime T cells. In the last 10 years, there has been an upsurge of data suggesting that dendritic cells (DCs) are the most important cells to stimulate immune responses against antigens [1]. DCs link the innate and adaptive immune responses by (i) binding a vast array of pathogens through their cell surface receptors, including C-type lectins, Toll-like receptors (TLRs), and scavenger receptors and inducing inflammatory responses for their elimination and (ii) are able to stimulate CD4+ T cell responses and cross present antigens for CD8+ T cell stimulation against antigens. Numerous strategies have been utilized to target antigens to DCs, following the abundance of information becoming available regarding cell surface expression of receptors and their role in stimulating immune responses [2]. The mannose receptor, DC-SIGN, scavenger receptor, DEC-205, and Toll-like receptors (TLRs) are amongst the most thoroughly studied DC receptors [2]. Targeting of these receptors is becoming an effective strategy of delivering antigens in DC-based anticancer immunotherapy studies.

TLRs are a class of proteins (pathogen recognition receptors, PRRs) that play a key role in the innate immune system and recognize molecules derived from pathogens (bacteria, fungi, virus, parasitic protozoa, mycoplasma), leading to stimulation of immune responses. Toll was first identified, almost 20 years ago, when it was found to have an essential role in the fly's immunity to fungal infections [3] and the first human TLR (TLR1) to be identified immediately followed [4]. Three years later, it was demonstrated that TLR4 initiated an adaptive immune response following ligation of the receptor with antibody [5], and lipopolysaccharide (LPS) was found to be the main ligand for TLR4 [6]. Using a series of gene ablations in mice, identification of other TLRs followed, mainly by Akira and colleagues [7–9], and to date 13, TLRs (TLR1-TLR13) have been identified. In brief, the ligands for each TLR are lipopeptides (TLR1), glycolipids, lipoproteins, heat shock protein (HSP)-70, zymosan (TLR2), double stranded RNA, poly I : C (TLR3), LPS, several HSPs (TLR4), flagellin (TLR5), multiple diacyl lipopeptides (TLR6), imidazoquinoline, loxoribine, bropirimine, imiquimod, resiquimod (TLR7), small synthetic compounds, imiquimod, resiquimod (TLR8), unmethylated CpG oligodeoxynucleotide DNA (TLR9), profilin (TLR11), and a bacterial ribosomal RNA sequence (TLR13). No ligands are known for TLR10 and TLR12. TLRs are expressed on different cells; however, all (except TLR12 which is exclusively expressed on neurons) are expressed on the key antigen presenting cells, monocytes, macrophages, DCs, and B cells. An exponential amount of papers are being published emphasizing the enhanced potency of vaccines by incorporating ligands that target TLRs on DCs with antigens, in animal models. TLR2 [10–12], TLR4 [13–18], TLR7 [19], TLR8 [20], and TLR9 [21] have been targeted with adjuvants which demonstrated significant antigen-specific enhancement in immune responses as compared to vaccinations without TLR agonists. 

IFN-gamma is a type II interferon produced by a variety of leukocyte populations including type I helper T (Th1) cells, natural killer (NK) cells, cytotoxic T lymphocytes (CTLs), antigen-presenting cells (APCs) including macrophages and DCs, and B cells. IFN-gamma is a potent immunomodulatory cytokine which exerts multiple biological effects on a range of cell types. Whilst typically known as an antiviral cytokine due to its capacity to block viral replication [22, 23], IFN-gamma has a broad range of functions on several arms of the immune system, including skewing T cell responses towards the type I helper T (Th1) cell phenotype [24, 25]. As a result, cellular immunity mediated by innate NK cells, adaptive CTLs, and macrophages [26]. IFN-gamma induces IL-12 and IFN-gamma production and inhibits IL-4 secretion and functions, resulting in suppression of the Th2 response [27–32]. These functional characteristics correspond evidently to its role in antimicrobial and antitumor immunity [33]. 

IFN-gamma priming has been shown to enhance macrophage activation through TLR ligation [34–37]. IFN-gamma promotes TLR ligand stimulation resulting in enhanced production of microbicidal nitric oxide and proinflammatory cytokines like IL-12. In addition to the synergy with TLRs, IFN-gamma alone enhances antigen processing and presentation in macrophages by upregulating subunits essential for the MHC-class I and II antigen presentation pathways [27–32]. Whilst the effect of IFN-gamma with or without TLR ligands on macrophages has been extensively studied, its adjuvanticity in DCs and its role in DC-mediated T cell proliferative responses have not been thoroughly clarified. In the current study, we investigate the effect of IFN-gamma on DC functional maturation and DC-meditated helper T cell activation, in the presence and absence of TLR ligation (TLR4 (LPS), TLR2/6 (zymosan) and TLR9 (CpG)). 

## 2. Material and Methods

### 2.1. Animals

C57BL/6 and OT-II mice (aged 6–10 weeks) used throughout this study were purchased from the animal facilities of the Walter and Eliza Hall Institute (Melbourne, Australia) or PAC in Alfred Medical Research and Education Precinct (AMREP), Melbourne, Australia. C57BL/6 mice were used as wild-type mice to evaluate IFN-gamma adjuvanticity. OT-II mice were donors of OVA helper peptide-specific CD4+ T cells. All mice were bred and maintained under specific pathogen-free conditions and were used in accordance with animal ethics guidelines. Ethics approval was granted by AMREP Ethics Committee, and all mice were treated and handled in accordance to the guidelines of the National Health and Medical Research Council (NHMRC) of Australia.

### 2.2. DC Generation and Purification

Bone marrow cells from femurs and tibias of C57BL/6 mice were collected by flushing with complete media (RPMI supplemented with 2% HEPES, 0.1 mM 2-ME, 100 U/mL penicillin, 100 *μ*g/mL streptomycin, 2 mM glutamine, and 10% FCS) through 70 *μ*M cell strainers and then were treated with red blood cell lysis buffer (0.15 M NH_4_Cl, 1 mM KHCO_3_, and 0.1 mM Na_2_EDTA) for 5 mins at room temperature. Washed cells were cultured in 24-well plates in complete media supplemented with 10 ng/mL GM-CSF and 10 ng/mL IL-4 (BD BioSciences, USA), at 5 × 10^5^ cells/well for 4-5 days. Cells were harvested by gently pipetting and were either used as is for proliferation assays (approximately 70% CD11c+) or were purified by magnetic cell sorting. Briefly, cells were pelleted and incubated with anti-CD11c MAC beads (400 *μ*L/10^8^ cells) (Miltenyi Biotec, Auburn, CA, USA) in the presence of 0.5% FCS and 2 mM EDTA in PBS at 4°C for 15 min. Cells were washed, resuspended, and purified using the autoMACS system (Miltenyi Biotec) following manufacturer's instructions. The percentage of CD11c+ cells purified in this manner was above 94% as measured by FACS analysis.

### 2.3. DC Maturation

To precondition DC for IFN-gamma studies, DC monolayers were incubated in complete media containing 10 ng/mL IFN-gamma for 2 hours. Cells were then washed and stimulated with either 1 *μ*g/mL LPS (derived from *Escherichia coli* (0111 : B4) Sigma, San Diego, USA), 20 *μ*g/mL zymosan A (from *Saccharomyces cerevisiae*, Sigma) or 10 *μ*g/mL CpG1668 (GeneWorks, Adelaide, Australia) for 16 h at 37°C. This procedure was previously optimized using the DC2.4 cell line (data not shown). Cells (5 × 10^5^) were washed and resuspended with FITC-conjugated anti-CD40 (FGK-45.5), anti-CD80 (16.10.A1), anti-CD86 (GL1), anti-MHC-class II (IA^b^) (M5/114.15.2), all constructed in house, or PE-conjugated anti-MHC-class I (BD BioSciences), together with APC-conjugated anti-CD11c (BD Biosciences) at 4°C for 30 min. Cells were then analyzed for expression of surface maturation markers by gating on live CD11c+ cells. 

### 2.4. T Cell Purification

Splenocytes from C57BL/6 or OT-II mice were collected, washed, and incubated in red blood cell lysis buffer at room temperature for 5 min. Cells were incubated with antibody mix which contained in-house produced rat anti-mouse Gr-1 (RB6-8C5), anti-CD11b (M1/70.15), anti-erythrocyte (TER-119), and anti-MHC-class II (M5/114.15.2) monoclonal antibodies at 4°C for 30 min. To purify CD4+ and CD8+ T cells, rat anti-mouse CD8-alpha (YTS169.4) and anti-CD4 (GK1.5) were included in the antibody mix, respectively. Labeled cells were depleted with 2 rounds of bead separation. In each round, cells were incubated with goat anti-rat Ig magnetic beads (8 beads/cell) (Qiagen, Melbourne, Australia) at 4°C for 25 min. Cells were washed and those that bound to the beads were removed by magnets. The purity of T cells was at least 94%.

### 2.5. Antigen-Specific T Cell Proliferation

Purified DCs were preconditioned with IFN-gamma (10 ng/mL) for 2 h and subsequently treated with endotoxin-depleted OVA (40 *μ*g/mL) and LPS (1 *μ*g/mL) or zymosan (20 *μ*g/mL) for 3 h. To evaluate the capacity of treated DCs to stimulate OVA-specific helper T cells, titrated DCs (1–4 × 10^3^) were seeded with 2 × 10^4^ purified OT-II CD4+ T cells in quadruplicates in 96-well plates. Proliferation of T cells was monitored by the addition of 1 *μ*Ci ^3^H-thymidine from day 1 to day 5. The radioactivity was measured in counts per minute (CPM). Peak proliferation of OT-II T cells on day 3 was compared. 

### 2.6. T Cell Costimulation Assay

C57BL/6 DCs (1–4 × 10^3^) pretreated with IFN-gamma and/or TLR ligands were seeded with 2 × 10^4^ C57BL/6 CD4+ T cells in quadruplicates in the 96-well plates precoated with 5 g/mL anti-CD3 (KT3-1.1). T cell proliferation was monitored by ^3^H-thymidine incorporation from day 2 to 7. Peak proliferation on day 5 was compared. 

### 2.7. *In Vivo* DC Maturation

C57BL/6 mice were injected with LPS (2 *μ*g) or CpG intradermally into each footpad, with or without IFN-gamma (2 ng). After 18 h, popliteal lymph node cells were collected. All mice were treated and handled as approved by the AMREP animal ethics committee, Melbourne Australia and in accordance to the ethics guidelines by NHMRC Australia. The maturation state of live CD11c+ DCs was determined by labelling with FITC-conjugated anti-CD80 and anti-CD86 and analyzed by flow cytometry. 

### 2.8. Statistical Analysis

All data are shown as the mean ± standard error of the mean (SEM). The data generated in this study were analyzed by student's *t*-test. Significance of difference was determined by the *P* value (≤0.05). 

## 3. Results

### 3.1. IFN-Gamma Enhances DC Maturation with or without TLR Ligands

**Figure 1:**
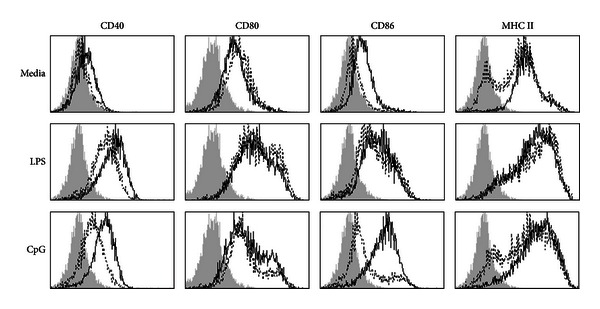
IFN-gamma enhances DC maturation with or without TLR ligands *in vitro*. C57BL/6 bone marrow cells were cultured with GM-CSF to generate bone marrow derived DCs. At days 4-5, cells were preconditioned with IFN-gamma for 2 h (solid line) or no IFN-gamma (dotted line), followed by LPS (TLR4 ligand) or CpG (TLR9 ligand) stimulation for 16 h. Cells were harvested and labelled with fluorescent antibodies. Live CD11c+ cells were gated and analyzed for CD40, CD80, CD86, and MHC-class II expression. Data shown are representative of at least two experiments. The shaded area represents cells stained with the respective secondary antibody.

The ability of IFN-gamma to promote DC maturation *in vitro* was assessed using day 5 bone marrow-derived DC in the presence or absence of TLR ligands, LPS (TLR4), and CpG (TLR9), by measuring cell surface expression of CD40, CD80, CD86, and MHC class II (Figure 1). IFN-gamma alone had a moderate effect on the upregulation of the activation markers, compared to untreated cells, most notably causing an enhancement in the levels of CD86 and MHC II expression. Likewise, CpG alone induced low levels of expression of the four surface markers compared to untreated cells; however, this was augmented in the presence of IFN-gamma, most notably, C40 and CD86. LPS strongly induced DC maturation as measured by the expression of the activation markers, and in the presence of IFN-gamma, only CD40 expression was further upregulated, albeit weak.

**Figure 2:**
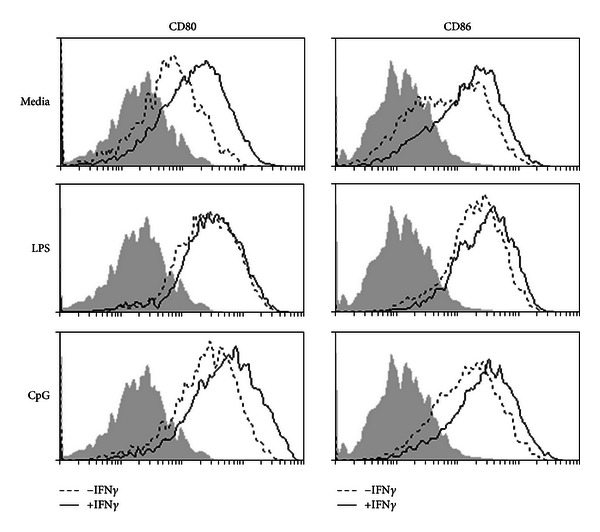
IFN-gamma enhances DC maturation with or without TLR ligands *in vivo.* C57BL/6 mice were injected with LPS (TLR4 ligand) or CpG (TLR9 ligand) with (solid line) or without (dotted line) IFN-gamma intradermally using the Hock immunization protocol. At 18 h, popliteal lymph cells were isolated. Live CD11c-high cells were analyzed for CD80 and CD86 expression. Data shown are representative of at least two experiments. The shaded area represents cells stained with the respective secondary antibody.

The ability of IFN-gamma to promote DC maturation *in vivo *was similarly assessed, following hock injection of mice with IFN-gamma in the presence or absence of TLR ligands (Figure 2). CD11c+ DCs from the popliteal lymph nodes showed increased CD80 and CD86 expression following IFN-gamma injection, compared to PBS-injected mice. Again, LPS alone strongly induced the expression of both activation markers which was not further augmented in the presence of IFN-gamma. CpG alone had minimal effect on CD86 expression, but increased CD80 expression; however, the inclusion of IFN-gamma further upregulated the expression of both markers, indicating enhancement of bone marrow-derived DC maturation.

### 3.2. IFN-Gamma Promotes DC Costimulation to CD4+ T Cells Only in the Presence of TLR Ligands

**Figure 3:**
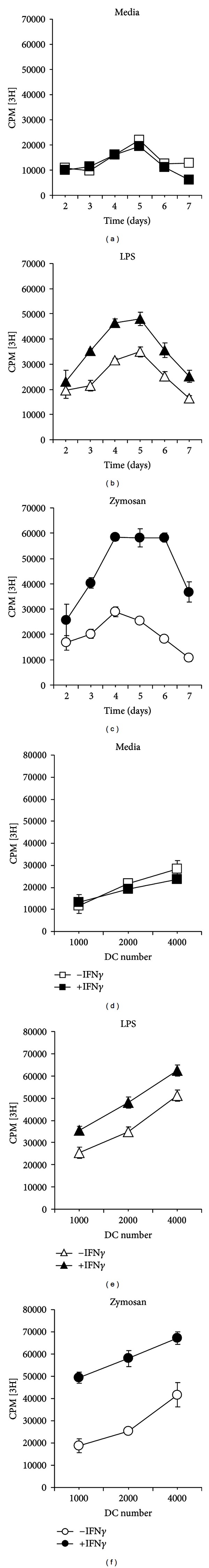
IFN-gamma enhances DC costimulation only when the TLR ligand is present. Days 4-5 bone marrow cultures preconditioned with IFN-gamma (black symbols) or no IFN-gamma (open symbols) for 2 h was stimulated with LPS (TLR4 ligand) or zymosan (TLR2 ligand) for 16 h. DCs were purified via the AutoMacs system as described in Section 2. Titrated bone marrow-derived DCs (1 × 10^3^–4 × 10^3^) were incubated with 2 × 10^3^ CD4 T cells in quadruplicates in 96-well plates that were precoated with anti-CD3. Cell proliferation was monitored from day 2 to day 7. Proliferation kinetics was exemplified when DCs were seeded at 2 × 10^3^ (a, b, c). As proliferation in general peaked at day 5, it was compared across DC titrations (d, e, f). Data shown are representative of two separate experiments. *P* < 0.05 in LPS and zymosan groups at all points except time point 2 days, based on quadruplicate values.

CD80 and CD86 which both bind CD28 and CTLA-4 on the surface of T cells providing regulatory signals leading to T cell activation are two of several cell surface molecules involved in T cell costimulation. Given the ability of IFN-gamma to upregulate surface expression of CD80 and CD86 on DC, we next investigated the capacity of these cells to promote T cell costimulation resulting in proliferation. Day 5 bone marrow-derived DCs were pretreated with IFN-gamma and TLR ligands, LPS, or zymosan and then assessed for their ability to co-stimulate proliferation of CD4+ T cells in the presence of immobilized anti-CD3 antibody (Figure 3). IFN-gamma-treated DCs alone were unable to induce CD4+ T cell proliferation, in line with the low levels of CD80 and CD86 expression observed on these cells (Figures 1 and 3). However, in the presence of TLR ligands, IFN-gamma-treated DC promoted a high level of CD4+ T cell proliferation, peaking at day 5. At this time point, the correlation between DC number and CD4+ T cell proliferation was assessed, with a positive trend between DC number and CD4+ T cell proliferation observed (Figure 3).

### 3.3. IFN-Gamma Enhances Antigen-Specific CD4+ T Cell Response Only in the Presence of TLR Ligands

**Figure 4:**
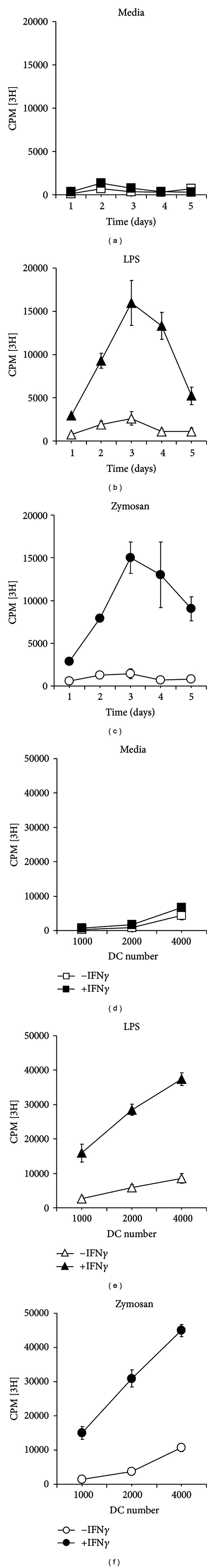
IFN-gamma enhances DC antigen presentation via MHC-class II, only in the presence of a TLR stimulus. Day 4 bone marrow cultures preconditioned with IFN-gamma for 2 h were pulsed with OVA in the presence of LPS (TLR4 ligand) or zymosan (TLR2 ligand) for 3 h. DCs were purified as described in Section 2. Titrated bone marrow-derived DCs (1 × 10^3^–4 × 10^3^) were incubated with 2 × 10^3^ CD4 T cells in quadruplicates in 96-well plates. Cell proliferation was monitored from day 1 to day 5. Proliferation kinetics was exemplified when DCs were seeded at 2 × 10^3^ (a, b, c). Peak proliferation at day 3 was compared across DC titrations (d, e, f). Data shown are representative of two separate experiments. *P* < 0.05 in all LPS and zymosan groups at all points, based on quadruplicate values.

The ability of IFN-gamma to potentiate antigen specific CD4+ T cell proliferation was investigated. DCs were incubated with IFN-gamma and pulsed with the model antigen ovalbumin (OVA) and then incubated with CD4+ transgenic T cells from OT-II mice which carry a transgenic CD4 T cell receptor specific for the MHC class II restricted OVA peptide, OVA_323–339_ [38]. The ability of the DC to induce proliferation of the OT-II CD4+ T cells in the presence and absence of TLR ligation was monitored from days 1–5 (Figure 4). Interestingly, the presence of TLR ligands alone induced CD4+ T cell proliferation to OVA very poorly. However, IFN-gamma pre-treatment dramatically enhanced antigen presentation by DCs, as evident with the high levels of CD4+ T cell proliferation. At the peak day of proliferation, day 3, the effect of DC number on proliferative responses was examined, with results again demonstrating a positive correlation between DC number and the magnitude of CD4+ T cell proliferation. 

## 4. Discussion

TLRs are essential receptors of the innate immune system which stimulate a vast array of inflammatory responses and eliminate invading pathogens. In addition, stimulation of TLR by binding to their respective ligands has been shown to lead to Th1, Th2, CD4+, and CD8+ T cell immune responses [39]. Antigens in combination with TLR ligand induce far superior immune responses compared to using antigen alone in animal models. Agonists to TLR7 activate plasmacytoid DCs (IFN-gamma, IFN-inducible protein, and IFN-inducible T cell alpha chemoattractant secretion), and TLR8 agonists activate myeloid DCs and monocyte-derived DCs (TNFalpha, IL-12, and MIP-1alpha, IFN-gamma) and upregulated CD40, CD80, and CD86 cell surface expression [40]. TLR7/8 agonists conjugated to HIV-1 Gag protein induce strong Th1/CD8+ T cell responses. Targeting TLR7 and TLR8 is effective in stimulating immune responses *in vivo* [41]. In TLR9 knockout mice, DCs stimulated with CpG have defective IL-12 and type-1 IFN secretion, even though Th1 and IFN-gamma responses were induced in TLR9 knockout mice following DNA immunizations [42]. TLR4 targeting has been shown to upregulate cell surface co-stimulatory markers (CD40, CD80, CD86), MHC molecules, and Th1 and Th2 cytokines on bone marrow-derived DCs [14–18]. Further, totally synthetic vaccines which target TLR2 (Pam3CysSer) carrying different antigens stimulate CD4+ and CD8+ T cell and/or antibody responses [10–12]. Targeting TLR5 using flagellin linked to antigens (ovalbumin (OVA), *Listeria monocytogenes* antigen p60 peptides or listeriolysin) induced IgG1, IgG2a antibodies, and protective CD8+ T cells responses in mice [43]. 

Phenotypic maturation and T cell stimulation are two functional attributes of DCs critical for immune induction, and their effective maturation into potent professional antigen presenting cells has been shown to be dependent on a number of critical cellular interactions, as well as by cytokine and TLR signalling. IFN-gamma is a key player in the development of T cell-mediated immunity and in mounting an adaptive immune response against infection or disease. In this study, we determined the ability of IFN-gamma to augment DC maturation and antigen presentation induced by TLR signalling. Data demonstrate that whilst IFN-gamma alone has a minor effect on DC functionality, however, when used to treat DC before subsequent TLR ligation, it significantly enhanced DC activation and T cell stimulatory capacity. 

In the present study, it is clear that IFN-gamma treatment of bone marrow-derived DC followed by incubation with the TLR4 (LPS) or TLR9 (CpG) agonists greatly enhanced DC activation compared to TLR ligation alone. Most notably, the upregulation of CD40 with LPS stimulation and CD86 with CpG stimulation was observed in *in vitro* cultures. Similarly, IFN-gamma coinjected with TLR ligands was able to promote DC activation *in vivo*, with DCs migrated from the site of immunization to the popliteal lymph nodes demonstrating increased expression of CD80 and CD86. The heightened DC activation translated to a drastic increase in T cell stimulatory capacity in both antigen independent and dependent fashions. This is the first time that IFN-gamma has been shown to have a combined effect with TLR ligation to enhance DC activation and function. In contrast, the effect of IFN-gamma on other APC populations has been well characterized. 

Much work has been done to study the effects of IFN-gamma treatment on macrophages, with the consensus of studies concluding that IFN-gamma primes macrophages into a semiactive state which is highly receptive to activation by a subsequent signal such as TLR ligation (for review see [44]). For example, upregulation of CD40 and CD80 on monocytes has been noted by IFN-gamma. Human acute myeloid leukemia blasts express low levels of both co-stimulatory molecules, demonstrating poor antigen presenting capacity. Incubation with IFN-gamma was found to up-regulate CD40 and CD80 expression, and this was found to be dependent on IRF-1 activation [45]. In addition, pre-treatment of macrophages with IFN-gamma induced pro-inflammatory cytokines, inducing an accumulation of IL-12 p40 and p35 mRNA, but only with subsequent TLR ligation by LPS was IL-12 protein produced [46]. However, more recent studies have demonstrated a cross talk between IFN-gamma and TLR signalling pathways, with multiple elements of the signalling pathways synergizing to induce expression of proinflammatory factors [47]. In DC, TLR engagement is an important factor in inducing DC maturation; however, as with macrophages, it is likely that a combination of TLR engagement and IFN-gamma signalling, thus mimicking the inflammatory conditions *in vivo*, is necessary to produce optimal DC activation. Indeed, the current studies show that the combination of both signals not only promotes the expression of activation markers but also corresponds with increased signalling to CD4+ T cells, in both nonspecific and antigen-specific fashions. 

Various signals can promote DC maturation, including direct cell-to-cell contact, cytokine signalling, and TLR signalling from microbial stimuli. Reports investigating the bidirectional cross talk between NK cells and DC have indicated that DC can activate NK cells which in turn enhance DC maturation [48]. In the presence of direct cell-to-cell contact, strong DC maturation was observed as indicated by CD86 expression; however, both IFN-gamma and TNF-alpha produced by the activated NK cells were found to enhance the levels of CD86 expression, although on their own the cytokines had little effect [48]. Likewise, in the current studies, IFN-gamma alone had little effect on the induction of DC maturation markers CD40, CD80, CD86, and MHC class II. In the presence of a secondary stimuli via TLR ligation, however, the upregulation of the cell surface markers was enhanced following IFN-gamma priming. 

In other studies, cultures of splenic DC with IFN-gamma upregulated expression of CTLA-4 counter receptor (however any counter receptor they measured in these studies is unclear), but not ICAM-1, heat stable antigen or MHC class I or class II [49]. However, despite the upregulation of this T cell receptor co-stimulatory signal, the ability of IFN-gamma treated DC to induce T cell proliferation was not enhanced. Similarly, another study investigating the effects of cytokine pre-treatment on DC function demonstrated that when DCs were cultured overnight with IFN-gamma and used in mixed lymphocyte reactions, the T cell proliferation was in fact lower than using untreated DC [50]. While the DC populations studied in these reports were different to the bone marrow-derived DC used in the current studies, the results clearly substantiate the current findings that additional costimulation (in the form of TLR ligation) is necessary to promote the adjuvanticity of IFN-gamma. 

The synergy between IFN-gamma and TLR ligands suggest that such combination is likely to be more highly beneficial to boost immune responses than IFN-gamma or TLR ligand alone in therapeutic settings for diseases, including cancer. Here, we unravel the adjuvant effect of IFN-gamma on DC maturation and T cell stimulation which are two important steps to achieve adaptive immunity for diseases, including cancer.
